# Histological Changes in Kidney and Liver of Rats Due to Gold (III) Compound [Au(en)Cl_2_]Cl

**DOI:** 10.1371/journal.pone.0051889

**Published:** 2012-12-27

**Authors:** Ayesha Ahmed, Dalal M. Al Tamimi, Anvarhusein A. Isab, Abdulaziz M. Mansour. Alkhawajah, Mohamed A. Shawarby

**Affiliations:** 1 Department of Pathology, College of Medicine, University of Dammam & King Fahd Hospital of the University, Al-Khobar, Saudi Arabia; 2 Department of Chemistry, King Fahd University of Petroleum and Minerals, Dhahran, Saudi Arabia; 3 Department of Pharmacology, College of Medicine, University of Dammam, Dammam, Saudi Arabia; Royal College of Surgeons, Ireland

## Abstract

**Introduction:**

Development of novel metallodrugs with enhanced anti-proliferative potential and reduced toxicity has become the prime focus of the evolving medicinal chemistry. In this regards, gold (III) complexes with various ligands are being extensively investigated. In the current study renal and hepatic toxicity of a newly developed gold (III) compound [Au(en)Cl_2_]Cl was assessed by histopathological evaluation of liver and kidney specimens of rats exposed to the compound.

**Methods:**

Male rats (n = 42) weighing 200–250 gram were injected single, varying doses of gold (III) compound [(dichlorido(ethylenediamine)aurate((III)]chloride [Au(en)Cl_2_]Cl in the acute toxicity component of the study. In the sub-acute toxicity part, a dose of 32.2 mg/kg (equivalent to 1/10 of LD50) was administered intraperitoneally for 14 consecutive days before sacrificing the animals. After autopsy, the renal and hepatic tissues were preserved in buffered formalin. Processing of the samples was followed by histopathological evaluation. The results were compared with the normal controls (n = 11).

**Results:**

A dose of 32.2 mg/kg (1/10 of LD_50_) revealed no renal tubular necrosis. The predominant histopathological finding was mild pyelitis, a prominence of eosinophils and mild congestion. The hepatic lesions comprised varying extents of ballooning degeneration with accompanying congestion and focal portal inflammation.

**Conclusion:**

Gold (III) compound [Au(en)Cl_2_]Cl causes minimal histological changes in kidney and liver of rats, reflecting its relative safety as compared to other clinically established antineoplastic drugs.

## Introduction

Gold is a noble metal and a commonly used material due to its oxidation resistance and unique electrical, magnetic, optical and physical characteristics. It exists in multiple oxidation states ranging from −1 to +5; the predominant form being Au (I) and Au (III) [1]. Metallic gold is known to be an inert and nontoxic metal. It is only the gold salts and radioisotopes that have pharmacological significance [1].

The use of gold compounds as medicinal agents is referred to as chrysotherapy [2]. Medical and therapeutic use of gold dates back to thousands of years [3]. In ancient cultures, around 2500 BC, gold was considered an integral component in the treatment of diseases such as measles, skin ulcers, and smallpox [4], [5]. In the 16^th^ century, gold was recommended for the treatment of epilepsy. Its rational medicinal use began in the early 1920’s when it was introduced as a treatment of tuberculosis [6]. Gold as an anti rheumatic agent was first reported in 1929 [7]. Gold and gold compounds are now mostly used for the treatment of various diseases including psoriasis, palindromic rheumatism, juvenile arthritis and discoid lupus erythematosus [8], [9]. However, following the body’s extensive exposure to gold compounds, it can diffuse to various organs like liver, kidney and spleen. Skin irritation, mouth ulcers, nephrotoxicity, liver toxicity and blood disorders have been associated with prolonged exposure to gold compounds [10].

Currently gold complexes have gained considerable attention due to their strong antiproliferative[11]–[14] and antiangiogenic potential [10]. The spectrum of gold complexes with documented cell growth inhibiting properties include a large variety of different ligands attached to gold in the oxidation states +1 or +3, that is gold (I) and gold (III) compounds [15], [16]. Gold (I) complexes proved to be unsuitable for clinical practice due to accompanying cardiotoxicity [17], [18], while studies on gold (III) complexes are comparatively scarce [8]. Gold (III) bears homology to cisplatin as it is isoelectronic with platinum (II) and tetracoordinate gold (III) complexes have the same square-planar geometries as cisplatin [3]. Cisplatin [*cis*-diamminedichloroplatinum(II)] is one of the most widely employed drugs in cancer chemotherapy, discovered more than 40 years ago [13], and it became the first FDA-approved platinum anticancer compound in 1978 [19]. Its effectiveness in solid tumoral lesions is markedly hampered by severe toxic side effects comprising predominantly nephrotoxicity [20], [21], development of tumor resistance[22]–[25] and occurrence of secondary malignancies [3], [12], [14] that contributes a high treatment failure ratio in clinical management.

Current studies aim towards designing newer compounds showing enhanced anti-proliferative potential and less associated toxicity than cisplatin. In this regards, gold (III) complexes with various ligands like Au–N, Au–S or Au–C bonds are being extensively investigated for their bioactivities as antiproliferative agents [26] and simultaneously new combinations of complexes are being developed. Milovanovic et al have studied the cytotoxicity studies of [Au(en)Cl_2_]^+^ and [Au(SMC)Cl_2_]^+^ where SMC = S-methyl-L-cysteine and [Au(DMSO)_2_Cl_2_]^+^ (DMSO = dimethyl sulphoxide). They concluded that gold (III) complexes are much faster to react with nucleophiles compare to Pt(II) complexes. They also demonstrated that gold (III) complexes exhibit relevant cytotoxic properties when tested on chronic lymphocytic leukemia cells (CLL). This conclusion indicates that gold(III) complexes have good potential for the treatment of cancer. In addition [Au(en)Cl_2_]^+^ complex shows cytotoxicity profiles comparable to cisplatin [27].

**Figure 1:**
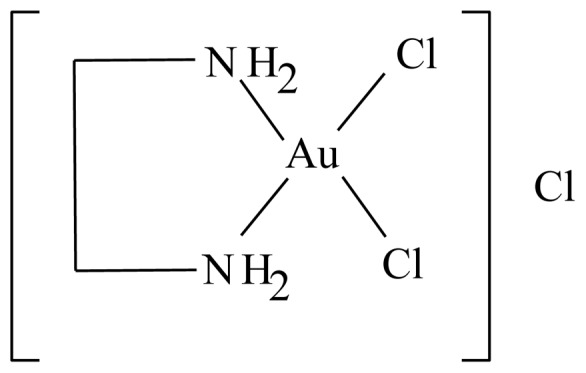
Dichlorido(ethylenediamine)-aurate(III) ion.

This study has led us to investigate further the conclusion achieved by the in vitro studies of Milovanovic et al [27]. The title compound is a newly developed gold (III) compound [Au(en)Cl_2_]Cl, gold complexed with N-substituted ethylenediamine. **(** **Fig.1** **).**

It has been prepared and fully characterized by spectroscopic techniques such as UV–Vis, Far-IR, IR spectroscopy, solution, X-ray and solid NMR. The solution NMR was measured in D2O, implicating that it is water soluble [28], [29]. In the current study we evaluated the histopathological toxicity of this compound in renal and hepatic tissues of rats.

## Materials and Methods

This study was carried out in Pathology Department, College of Medicine, University of Dammam in 2010–2011. It was compartmentalized into two segments comprising acute toxicity and subacute toxicity studies. For both segments, Albino Wistar male rats (n = 42), weighing 200–250 gram were obtained from the College of Veterinary Medicine, King Faisal University, Al-Hassa, Saudi Arabia. They were placed in an animal house under standardized conditions, fed standard chow and exposed to an optimized environment one week before the start of the experiment.

### Acute Toxicity Study

In acute toxicity, 5 groups of rats (A/I-E/I), with each group comprising 5 animals, were administered gold compound intraperitoneally in doses of 1500 mg/kg, 750 mg/kg, 375 mg/kg, 187.5 mg/kg and 93.75 mg/kg, respectively. A control group of 5 animals (F/I) was simultaneously administered 0.2 ml water intraperitoneally.

After 24 hours, the number of deceased rats was counted in each group and LD_50_ (dose that kills 50% of animals) was calculated (322 mg/kg) by the method of Miller and Tainter [30].

Autopsy was carried out in all animals and renal as well as hepatic tissues were preserved in 10% buffered formalin for subsequent evaluation of histopathological alterations.

### Sub-acute Toxicity Study

The rats in this component of the study were divided into two treatment groups, A/II and B/II, with six rats in each. Group “A/II” served as the experimental group while group “B/II” served as the control. Rats in the experimental group (A/II) were injected with 32.2 mg/kg (1/10 of LD_50_) body weight of the gold compound while rats in the control group (B/II) were injected with normal saline daily for 14 days.

Autopsy was carried out in all the rats. Renal and hepatic tissues were preserved in 10% buffered formalin until subjected to histopathological evaluation.

### Histopathological Work Up

#### a) Fixation and tissue processing

The formalin preserved hepatic and renal tissue samples of [Au(en)Cl_2_]Cl dosed rats and controls were processed in an automated tissue processor (Tissue–tek VIP-5, from SAKURA). The processing consisted of an initial 2 step fixation comprising tissue immersion in 10% buffered formalin for two hours each, followed by removal of fixative in distilled water for 30 minutes. Dehydration was then carried out by running the tissues through a graded series of alcohol (70%, 90%, and 100%). The tissue was initially exposed to 70% alcohol for 30 minutes followed by 90% alcohol for 1 hour and then two cycles of absolute alcohol, each for one hour.

Dehydration was then followed by clearing the samples in several changes of xylene. It consisted of tissue immersion for an hour in a mixture comprising 50% alcohol and 50% xylene, followed by pure xylene for one and a half hour. Samples were then impregnated with molten paraffin wax, then embedded and blocked out. Paraffin sections (4–5 um) were stained with hematoxylin and eosin, the conventional staining technic [31].

Stained sections were examined for necrosis, apoptosis, inflammation and vascular changes in renal tissue.

The hepatic tissue was evaluated for any alterations in the architecture, portal or lobular inflammation, sinusoidal dilatation and congestion along with presence of granulomas, degeneration, necrosis and fatty change.

#### b) Histopathological grading for renal lesions

Renal lesions in [Au(en)Cl_2_]Cl dosed rats were assessed by light microscopy and graded into five categories by utilizing a scale of 0 to 5 as mentioned and adopted by Zhang et al [32]:

**0** =  normal histology,

**1** =  tubular epithelial cell degeneration, without significant necrosis/apoptosis;

**2–5** =  <25%, <50%, <75% and >75% of the tubules showing tubular epithelial cell necrosis/apoptosis, respectively, accompanied by other concomitant alterations.

#### c) Histopathological categorization of hepatic lesions

The hepatic lesions were categorized according to the criteria mentioned below by Ramchandran et al [33] **(** **table 1** **)**.

**Table 1:** Histological categorization of drug-induced hepatic lesions.

Acute hepatitis and cholestatic hepatitis	
Acute liver failure	Necrosis with marked inflammation, Necrosis with little or no inflammation
Cholestasis	Bland cholestasis, Cholestatic hepatitis
Chronic Hepatitis	
Granulomatous hepatitis	
Steatosis/Steatohepatitis	Macrovesicular, Microvesicular, Steatohepatitis
Vascular Abnormalities	Sinusoidal obstruction syndrome

## Results

**Figure 2:**
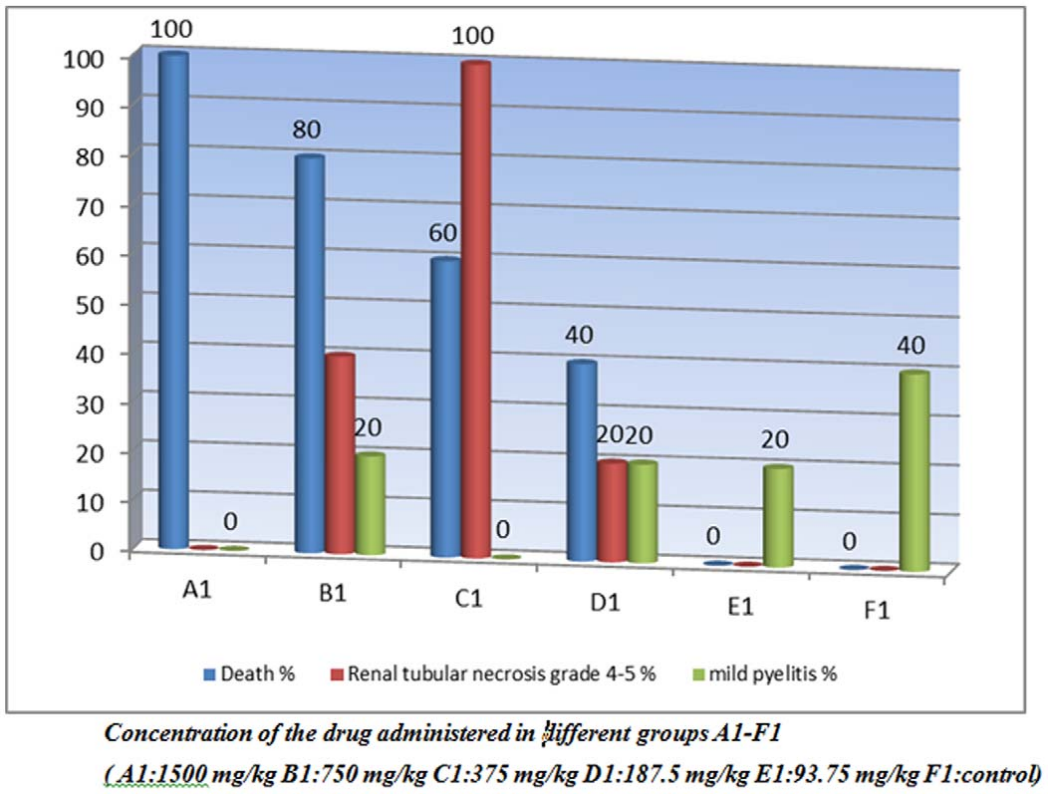
Spectrum of renal tubular necrosis seen in acute toxicity study of a gold (III) compound [Au(en)Cl_2_]Cl.

*The results of the study are depicted in* *tables 2* *,* *3* *,* *4* *and* *Figures 2* *,* *3* *,* *4* *,* *5* *,* *6* *,* *7* *, -8.*

**Figure 3:**
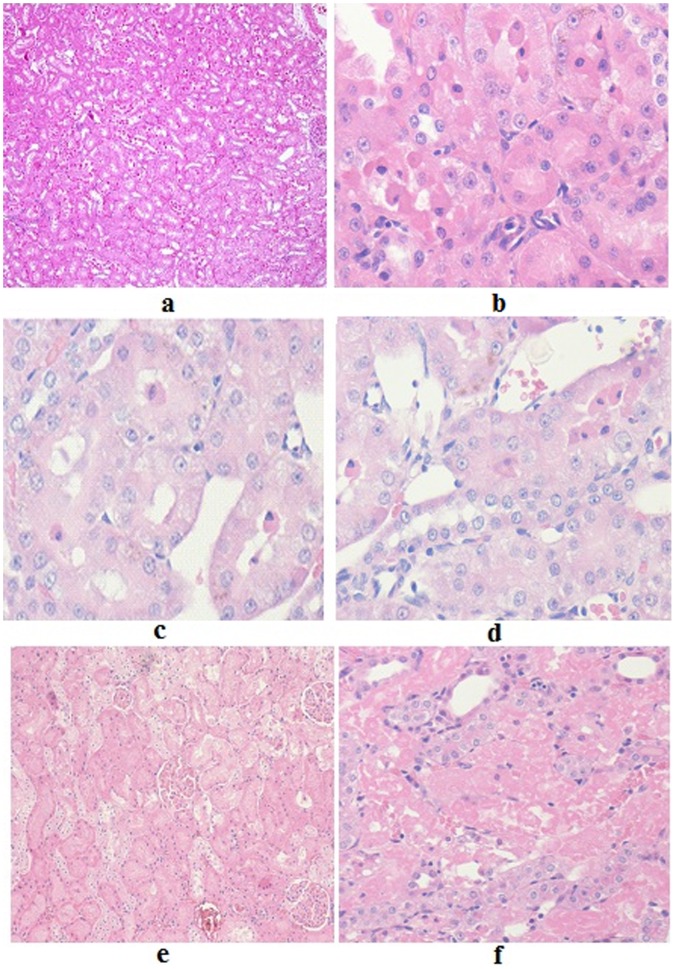
Microscopic findings of renal tubules showing different grades of renal tubular necrosis as seen in the acute toxicity study of a gold (III) compound [Au(en)Cl_2_]Cl. **a & b:** Grade 2 as seen in H&E ×20 and ×40. Necrotic tubules are seen amongst viable renal tubules. The necrosis is less than 25% of the total material examined. In ×40 magnification, more abundant, necrotic cells are seen along with normal renal tubules. **c & d:** Grade 1 as seen in H&E ×40 magnification. Scattered individual apoptotic/necrotic cells with strongly eosinophilic cytoplasm and pyknotic nuclei are seen. **e & f:** Grade 5 as seen in H&E ×20 and ×40 The entire field shows mostly necrotic renal tubules.

**Figure 4:**
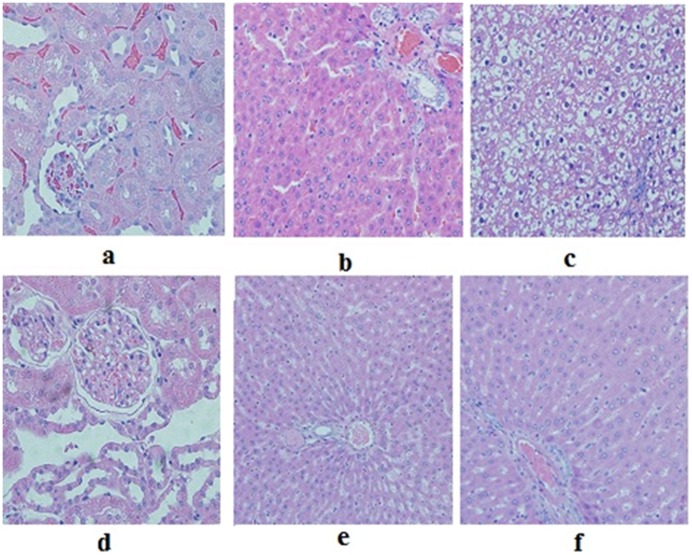
Renal and hepatic tissues in the controls used in acute (a,b,c) and sub-acute (d,e,f) toxicity parts of study. a: Renal tissue showing mild congestion with no other pathological change as seen in acute toxicity controls (H&E x40). **b:** Hepatic tissue as seen in acute toxicity controls (H&E x40) showing mild congestion. No other pathological change is seen in this focus. **c:** Marked ballooning degeneration as seen in acute toxicity controls (H&E x40). **d:** Unremarkable renal tubules as seen in sub-acute toxicity controls (H&E x40). **e:** Unremarkable hepatic tissue as seen in sub-acute toxicity controls (H&E x20). **f:** Unremarkable hepatic tissue as seen in sub-acute toxicity controls (H&E x40).

**Figure 5:**
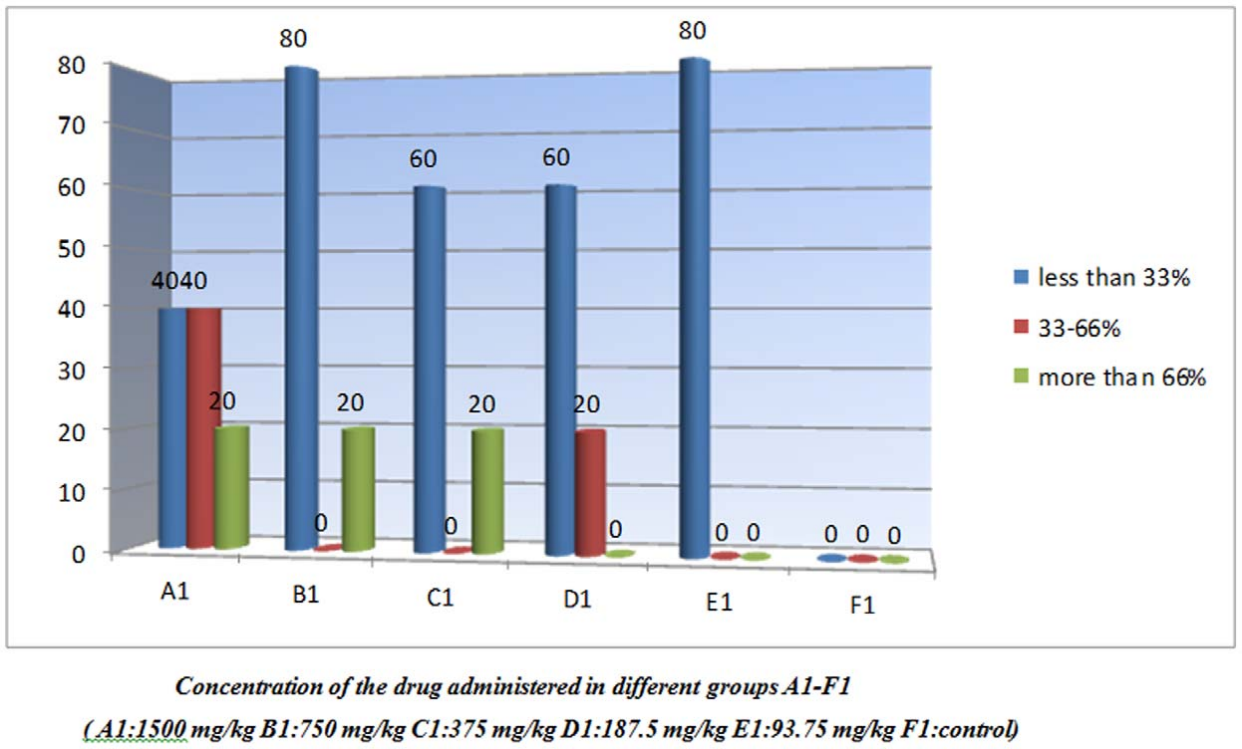
Extent of hepatic steatosis seen in acute toxicity study of a gold (III) compound [Au(en)Cl_2_]Cl.

**Figure 6:**
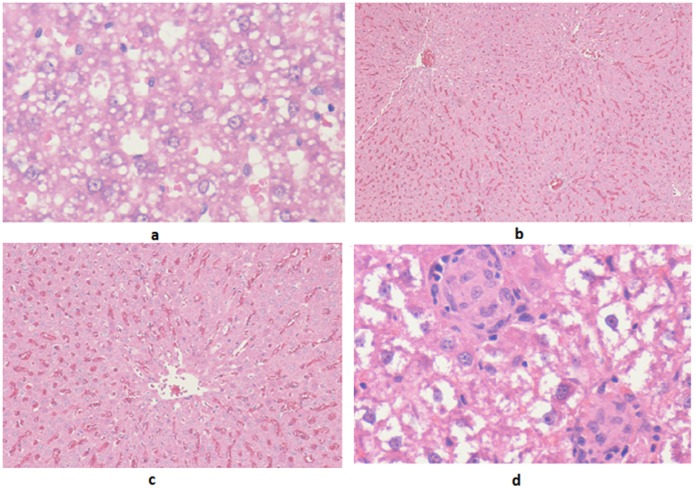
Spectrum of hepatic microscopic findings as seen in the acute toxicity study of a gold (III) compound [Au(en)Cl_2_]Cl. a: Marked mixed micro and macrovesicular steatosis, H&E ×40. b & c: Marked sinusoidal congestion and dilatation, H & E ×20 and ×40 respectively. d: Marked ballooning degeneration along with two microgranulomas, H & E ×40.

**Figure 7:**
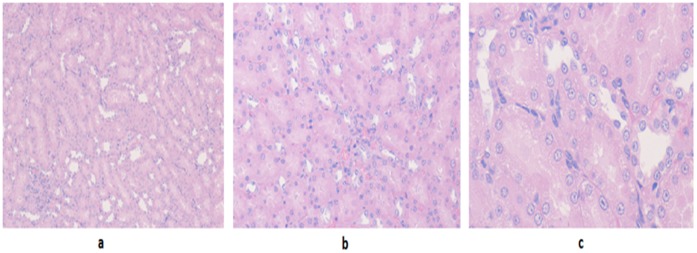
Microscopic pictures of renal tubules, with no evidence of necrosis as seen in sub-acute toxicity study of a gold (III) compound [Au(en)Cl_2_]Cl, H&E at magnifications of : a. ×10. b. ×20. c. ×40.

**Table 2:** Acute toxicity, salient hepatic microscopic findings.

Groups	Ballooning degeneration			Hepatocellular necrosis/degeneration		Sinusoidal Obstruction syndrome	Inflammation portal/lobular			Congestion	
	Mild	Mod	marked	Individual cell degeneration	Necrosis with inflammation		Mild	Mod	Marked	Mild	Mod/marked
A/I (n = 5)	–	–	–	20% (1)	–	80% (4)	60%(3)	–	–	–	–
B/I (n = 5)	–	–	–	40% (2)	–	20% (1)	20%(1)	–	–	–	80%(4)
C/I (n = 5)	–	–	–	–	–	–	-	–	–	40%(2)	60%(3)
D/I* (n = 5)	–	–	–	–	–	–	40%(2)	–	–	–	100% (5)
E/I ** (n = 5)	–	20%(1)	–	–	20%(1)	–	20%(1)	–	–	20%(1)	60%(3)
F/I (n = 5)	–	60%(3)	40% (2)	–	–	–	40%(2)	–	–	20%(1)	40%(2)

* Capsular inflammation with peritonitis was discerned in 40% of cases.(fibrinopurulent exudates on the surface).

** An occasional microgranuloma was present in 20% of cases.

**Table 3:** Sub-acute toxicity, salient renal microscopic findings.

Groups (n = 6 in each group)	Dosage mg/kg	Death %	Pyelitis/interstitial inflammation		Congestion	
			Mild	Mod/marked	Mild	Mod/Marked
**A/II (n = 6)**	32.2	0	100% (6)	–	100% (6)	–
**B/II (n = 6)**	0	0	83.33% (5)	16.66% (1)	100% (6)	–

**Table 4:** Sub-acute toxicity, salient hepatic microscopic findings.

Group	Dosage mg/kg	Death%	Ballooning degeneration			Inflammation portal/lobular			Congestion	
			Mild	Moderate	Marked	Mild	Moderate	Marked	Mild	Mod/marked
**A/II** ** **/** *** **(n = 6)**	32.2	0	16.66% (1)	16.66% (1)	66.66% (4)	83.33% (5)	–	–	83.33% (5)	16.66% (1)
**B/II (n = 6)**	0	0	16.66% (1)	16.66% (1)	16.66% (1)	66.66% (4)	–	–	33.33% (2)	66.66% (4)

** 100% (6) cases revealed capsular inflammation.

*** 16.66% (1) case revealed an occasional microgranuloma.

### Acute Toxicity

#### Renal Microscopic Findings

The renal lesion in all groups of this batch demonstrated variable extent of renal tubular necrosis/apoptosis (Fig. 2) with one grade showing slight predominance over the other. No single group specific necrosis grade was evident in the entire series.

All the 5 rats in group A/I (Dose: 1500 mg/kg) died before sacrificing. The renal microscopy revealed normal histology in three animals and tubular necrosis of grade 2 severity i.e. comprising less than 25% of the total tubular tissue, in the remaining two cases (Fig. 3a and 3b). Scattered occasional tubules with vacuolated cytoplasm were also seen along with one of the case showing cells with strongly eosinophilic cytoplasm.

In group B/I (Dose: 750 mg/kg), four out of five animals died before sacrificing. Again, a large range of necrosis was discerned, with three animals revealing grade 1 (Fig. 3c and 3d), one grade 4 and the last grade 5 tubular necrosis.

In group C/I (Dose: 375 mg/kg), three out of five animals died before sacrificing. All animals showed renal tubular necrosis comprising 75% or more of the total renal tissue examined (grade 5, Fig. 3e and 3f).

Group D/I (Dose: 187.5 mg/kg) had two dead animals out of five, before sacrificing. A wide range of renal tubular necrosis comprising around 25% to more than 75% of total tissue (predominantly grade 2) was discerned.

Group E/I (Dose: 93.75 mg/kg) with all 5 animals alive at necropsy, revealed renal tubular necrosis varying in range from individual cell necrosis/apoptosis to necrosis constituting less than 50% of the total renal tissue examined (predominantly grade 2–3).

The control group (F/I) with all animals alive revealed normal renal tubular histology (Fig. 4a).

Varying extent of congestion dominated the entire histopathological spectrum.

#### Hepatic microscopic findings

The hepatic specimens of almost all 5 animals of each group, A/I, B/I, C/I, D/I and E/I revealed variable extent of micro and macro-vesicular steatosis (Fig. 5 and Fig. 6a). Varying extent of congestion (Fig. 6b and 6c) along with few cases showing sinusoidal obstruction syndrome were also present. In A/I and B/I, one and two cases respectively, revealed scattered individual hepatocytic cell degeneration without inflammation. One case showing focal necrosis with inflammation and another one revealing moderate ballooning degeneration with an occasional microgranuloma was seen in group E/I (Fig. 6d). The hepatic picture in F/I (control, drug free group, Fig. 4b and 4c) comprised moderate to marked ballooning degeneration (percentages of hepatic lesions are shown in table 2).

### Sub-Acute Toxicity

This batch had two groups, each comprising 6 animals. The first group (A/II) was dosed with 32.2 mg/kg (1/10 of LD_50)_ for two weeks and the second (group B/II) was the drug free control group.

**Figure 8:**
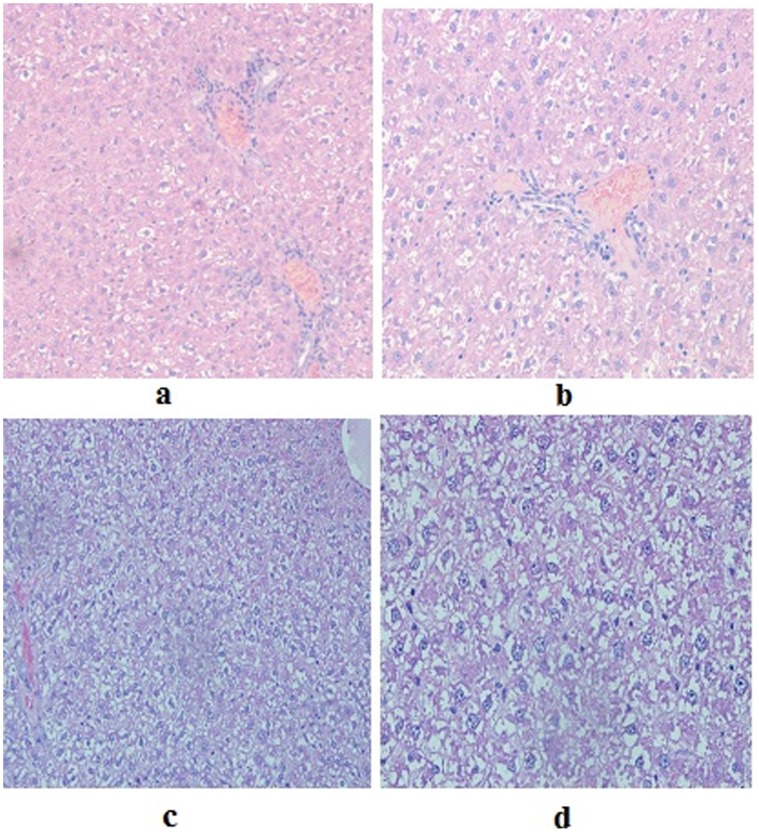
Hepatic microscopic findings in sub-acute toxicity study of a gold (III) compound [Au(en)Cl_2_]Cl. a: Mild ballooning degeneration, H&E ×20. **b:** Mild ballooning degeneration, H&E × 40. **c:** Marked ballooning degeneration, H&E ×20. **d:** Marked ballooning degeneration, H&E ×40Toxicity.

Group A/II had no animal dead before necropsy. As a whole, the renal tissue was unaffected as far as tubular necrosis (Fig. 7) was concerned. Varying extents of pyelitis with prominence of eosinophils and mild congestion spanned the entire histological picture (percentages are shown in table 3). The hepatic lesion comprised mild to marked ballooning degeneration (Fig. 8) and congestion, with one case revealing an occasional microgranuloma. Capsular inflammation, focal portal inflammation and an occasional focus of lobular inflammation completed the entire histological spectrum (percentages are shown in table 4).

In Group B/II the renal histology was within normal limits (Fig. 4d) with pyelitis, congestion and focal pigment deposition constituting the consistent microscopic findings (table 3). The hepatic picture ranged from normal, unaffected liver (Fig. 4e and 4f) in three cases to mild, moderate and marked ballooning degeneration, respectively, in the remaining three cases in this group (table 4). No steatosis was present in animals of this group.

## Discussion

This study demonstrated minimal renal and hepatic toxicity by a newly developed gold (III) compound, [Au(en)Cl_2_]Cl. In the sub-acute toxicity part of the study, this compound showed dose dependent renal toxicity but with a much extended nephrogenic safety range and also exhibited a notably higher safe upper limit compared to toxicity levels of clinically established antineoplastic drugs like cisplatin, doxyrubicin and 5-Florouracil(5-FU) as reported in other studies. Comparative analysis with other gold compounds was limited by paucity of toxicity studies. Many studies report gold(III) complexes as emerging, potential anticancer agents [34], [35], [36], [37] with elaboration of their mechanisms of action and antiproliferative activity [27], [35] against many different cancer stem lines, but their toxicity data as regards detailed renal and hepatic histopathological manifestations have not been adequately described.

In our study a dose of 32.2 mg/kg (1/10 of LD_50_) revealed normal renal tubular histology with no evidence of tubular necrosis. Mild pyelitis with a prominence of eosinophils and mild congestion was a consistent finding. Varying extent and grade of renal tubular necrosis was only seen with the administration of the gold(III) compound at very high dosages (range of 187.5–1500 mg/kg), administered in the acute toxicity component of the study.

Other antineoplastic drugs are seen to exhibit a significantly low renal tolerance. In a study comprising multi drug analysis by Hanigan et al, rats dosed intraperitoneally with 15 mg/kg of body weight cisplatin revealed grade 4 tubular necrosis [38]. Atasyara et al described remarkable epithelial vacuolation, necrosis, and desquamation of cells with protein casts in renal tubules after a single intraperitoneal dose of 7.5 mg/kg of cisplatin [39].In a study by Ravindra et al, rats injected intraperitoneally with 0.4 mg/kg of cisplatin for a period of 8 weeks showed different alterations comprising marked proximal tubular dilation and desquamation along with acute tubular necrosis [40]. Other drugs like methrotrexate and cyclosporine have been reported to have a nephrotoxic effect culminating to cell death by direct tubular toxicity and intratubular precipitation [41], [42] along with proximal tubular apoptosis and necrosis [43] respectively, but studies evaluating their dose dependent renal histopathological manifestations are not available.

Nephrotoxicity is an integral and inherent accompaniment of multiple anti-neoplastic drugs [23], [24], [44]–[46] which usually have a narrow therapeutic index and the minimum dosage required to significantly decrease tumor burden is usually associated with substantial nephrotoxicity. The significantly diminished renal toxicity of N-substituted ethylenediamine complexes of gold could be attributed to their different anti-proliferative mechanism of action and selective sparing of the proximal tubular epithelial cells. Their mechanism although not precisely delineated, comprises a cumulative impact on induction of cell cycle blockage, interruption of the cell mitotic cycle, programmed cell death (apoptosis) or premature cell death (necrosis) [47].

Hepatotoxicity is an entity not as extensively explored as nephrotoxicity as it does not manifest itself as a dose limiting factor [48]. With our ethylenediamine derivative of gold, in the acute toxicity component of the study, varying extent of steatosis was the main finding. In the sub acute toxicity component, varying extent of ballooning degeneration with accompanying congestion and focal portal inflammation comprised the predominant histopathological lesion. One of the samples revealed an occasional focus of lobular inflammation. Capsular inflammation was also a consistent finding. Other drugs like cisplatin produce hepatoxicity in high doses [49], [50]. El-Sayyad et al investigated the effects of cisplatin, doxorubicin and 5-FU belonging to different chemical classes on rats liver and showed that groups receiving cisplatin and doxorubicin exhibited increased hepatoxicity in comparison to 5-FU treatment. The most pronounced histopathlogical abnormalities observed were hepatic cord dissolution [51]. Avci et al demonstrated that a dose of 10 mg/kg cisplatin could induce sinusoidal congestion, hydropic and vacuolar degeneration, extensive disorganization in hepatocytes, and significant fibrosis around central venules and expanded periportal areas [48]. In another multidrug, multimodal study by Kart et al, moderate to severe hydropic degeneration in centrilobular zones extending towards the portal region was obtained with a single intraperitoneal 6.5 mg/kg dose of cisplatin. Necrotic hepatocytes, especially concentrated around the central veins, were observed in the severely affected cases [52].

Ballooning degeneration was a finding that was also evident in the control group of animals as well. As regards ballooning degeneration, the non significant difference between controls and drug dosed rats in hepatic toxicity in the sub-acute group reflects that drug toxicity may not be the only reason for the hepatic lesion.

The hepatic lesion produced by N-substituted ethylenediamine complexes with gold was substantially milder than cisplatin with no evidence of apoptosis or necrosis in the entire series of animals receiving a drug dose of 32.2 mg/kg for 14 days.

### Conclusions

Gold (III) compound [Au(en)Cl_2_]Cl in sub-acute toxicity study, produced less renal and hepatic toxicity as compared to other clinically established antineoplastic drugs. In the entire series of animals, no renal tubular necrosis was seen. Mild pyelitis and congestion dominated the histopathological picture. In hepatic tissue, ballooning degeneration of varied extent and severity prevailed in the drug dosed animals with no evidence of hepatocytic degeneration and necrosis.
